# Retronychia: Surgical treatment and postoperative care

**DOI:** 10.1111/ddg.70202

**Published:** 2026-03-19

**Authors:** Galina Balakirski, Alexander Kreuter, Silke C. Hofmann, Thorsten Neubert, Alexandros Paschos

**Affiliations:** ^1^ Center for Dermatology, Allergology and Dermatosurgery Helios University Hospital Wuppertal University Witten/Herdecke Wuppertal Germany; ^2^ Department of Dermatology, Venerology and Allergology Helios St. Elisabeth Hospital Oberhausen University Witten/Herdecke Oberhausen Germany; ^3^ Department of Dermatology, Venerology and Allergology Helios St. Johannes Hospital Duisburg Duisburg Germany

**Keywords:** avulsion, distal nail embedding, Howard‐Dubois technique, kinesiology tape, nail surgery, Retronychia, taping

## INTRODUCTION

The term retronychia describes the ingrowth of the proximal regions of the nail plate into the proximal nail fold.^1^ Clinically, this results in chronic paronychia at the proximal nail fold, a thickening of the proximal part of the nail plate and an interruption of nail growth. Typically, the big toes are affected, either unilaterally or bilaterally. In terms of pathogenesis, an excessively long toenail with consecutive trauma, repeated trauma or microtrauma, for example through sports or wearing uncomfortable, tight shoes, play the most important role.^1^, ^2^, ^3^ In a traumatic event, the nail plate detaches completely from the nail matrix. A new nail plate grows under the old one, pushes it upwards, and itself is pressed into the proximal nail fold. This results in several nail plate generations, which are objectified in retronychia.^1^, ^2^

The exact prevalence of the disease is unclear. Reports suggest that the symptoms are often misinterpreted as a bacterial infection or onychomycosis and that it can take several months to years to diagnose and treat the condition.^4^ Although conservative therapies such as detachment of the hyperkeratotic nail sections with urea‐containing topical agents, podiatric treatment with grinding of the thickened nail plate down to the final nail layer close to the nail bed, topical and intralesional steroids and application of tape dressings can lead to relief of symptoms,^3^, ^5^, ^6^ proximal, surgical nail avulsion is the most effective treatment option with the lowest recurrence rates (between 10 and 21.8%).^7^, ^8^ However, it takes several months for the new toenail to regrow completely after avulsion due to the physiologically slow growth rate of the toenails. During this entire time, the nail bed requires consistent care to allow the regrowth of the new, healthy nail. In addition, the arching (hypertrophy) of the hyponychium in the absence of a nail plate can lead to so‐called “distal embedding” of the nail, the painful ingrowth of the distal nail edge into the distal nail fold. Surgical treatment of retronychia therefore requires good cooperation from the patient to achieve an optimal outcome. Comprehensive patient education and regular post‐operative follow‐up checks are important in the treatment of retronychia.

Using two cases of retronychia as examples, we illustrate possible postoperative courses with subsequent treatment and therapy results.

## CASE PRESENTATION AND SURGICAL TECHNIQUE

### Case 1

A 39‐year‐old female patient had been suffering from painful retronychia with chronic paronychia and granulation tissue on the proximal nail fold of the right big toenail for about four months (Figure 1a). A few years previously, she had undergone an “Emmert plasty” for an ingrown toenail of the same big toe. We performed a minimally invasive proximal avulsion (Figure 1b). At the beginning a regular nail growth could be observed. However about three months after avulsion a significant hypertrophy of the distal nail fold occured. Thus taping of the distal nail fold was initiated (Figure 1c). However, five months after avulsion, painful distal embedding of the nail occurred (Figure 1d). After thoroughly informing the patient, a further conservative approach was chosen. Daily moisturizing nail bed care was continued and kinesiology tapes were applied by the patient to alleviate the hypertrophy of the distal nail fold (Figure 1e,f). After twelve months, the nail growth was unremarkable (Figure 1g).

**FIGURE 1:**
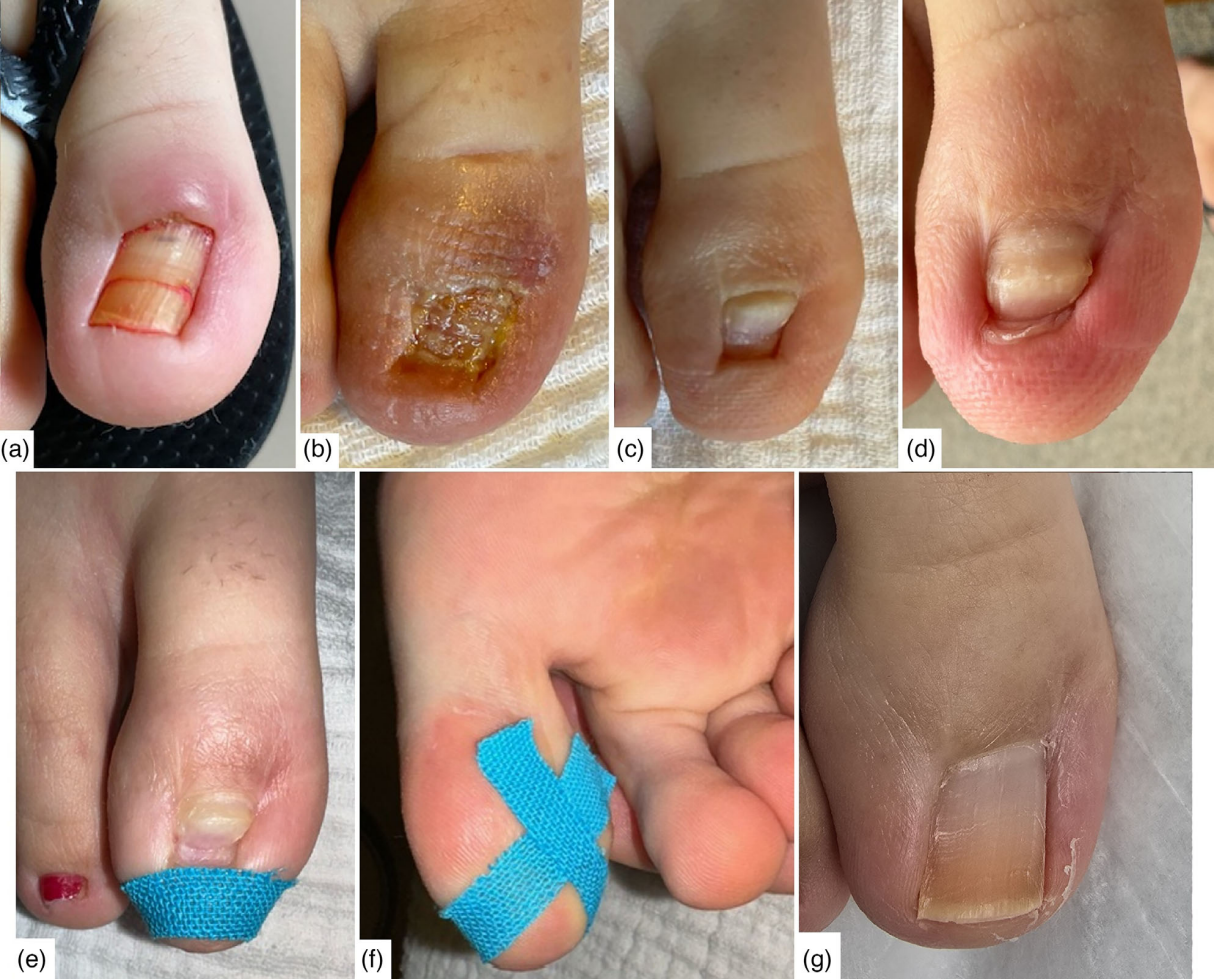
Clinical presentation of retronychia with inflammation at the proximal nail fold, discoloration of the nail, and visible scars after previous surgery (a). Clinical findings two days after avulsion (b). Initial nail growth three months after avulsion with already noticeable slight hypertrophy of the distal nail fold (c). Significant hypertrophy of the distal nail fold with initial distal nail embedding of the distal nail edge five months after avulsion (d). Application of kinesiology tape to the distal nail fold to prevent further hypertrophy, ventral (e) and dorsal view (f). Nail appearance one year after avulsion with regular nail growth (g).

### Case 2

A 40‐year‐old female patient had been suffering from painful retronychia with chronic paronychia at the proximal nail fold of the left big toe for about five months (Figure 2a). A minimally invasive proximal avulsion was performed (Figure 2b). Initially, there was a regular nail growth (Figure 2c). After six months, however, painful ingrowth of the distal nail edge occurred with hypertrophy of the distal nail fold (Figure 2d). Due to impatience, the patient refused the application of the kinesiology tapes. In order to correct the hypertrophy of the distal nail fold and to allow the nail to grow out, a so‐called “fish mouth plasty” was performed (Figure 2e,f). After only three months, the nail growth was unremarkable (Figure 2g).

**FIGURE 2:**
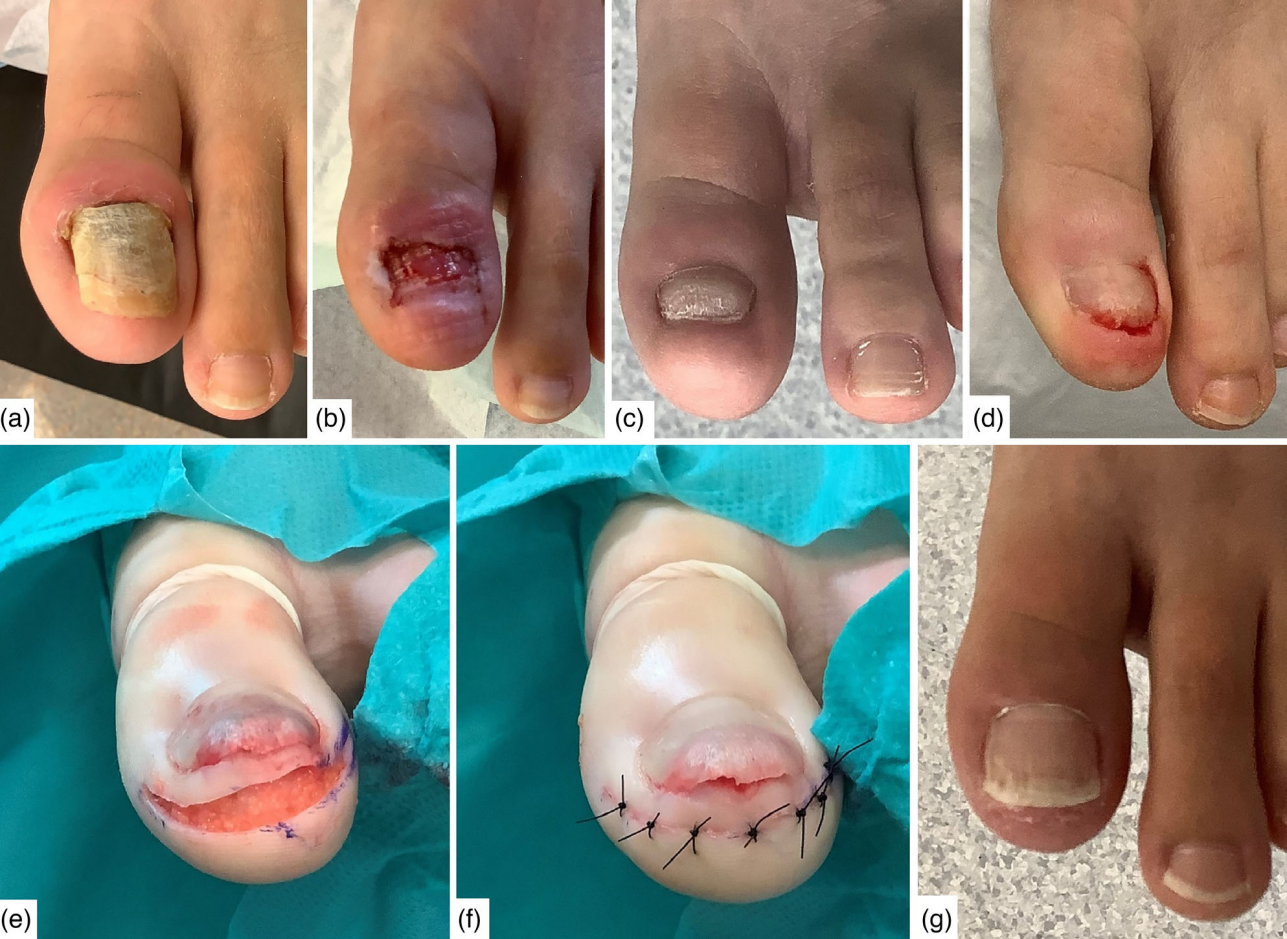
Clinical presentation of retronychia with inflammation at the proximal nail fold, as well as thickening and discoloration of the toenail (a). Clinical findings a few days after avulsion (b). Initial nail growth three months after avulsion with initial hypertrophy of the distal nail fold (c). Painful distal nail embedding of the distal nail edge with marked hypertrophy of the distal nail fold (d). Intraoperative presentation of the “fish mouth plasty”a to reduce the hypertrophy of the distal nail fold with the incision approximately 0.5 cm below the hyponychium (e) and findings immediately after surgery (f). Nail condition 10 months after avulsion with regular nail growth (g).

## DISCUSSION

There are currently several case reports and case series on the treatment of retronychia.^4^, ^5^, ^6^, ^7^, ^8^ Although efforts have been made to establish an effective conservative treatment for retronychia, proximal avulsion is still considered the gold standard and has the lowest recurrence rate.^9^ In a prospective study, 20 patients with retronychia were treated conservatively by milling off the damaged nail down to the last nail layer close to the nail bed.^6^ The sessions were repeated every 7 to 14 days, and for hypertrophy of the distal nail fold, additional tape dressings were applied. The duration of therapy varied from 2 to 14 months, and not a single relapse was observed after a total of 18 months. The method is effective, but also relatively time‐consuming, and it requires several sessions.^6^ In a multicenter study of 42 patients with retronychia, avulsion was superior to conservative therapy, with a recurrence rate of around 10% versus recurrence rate of 30% for conservative treatment.^7^ A systematic review from 2022 analyzed 231 patients from 24 studies. Conservative therapy was found to lead to healing in only 41.2% of cases, though it was very well tolerated.^8^ Proximal avulsion showed the best remission rates of 78.2%, but was associated with long‐lasting complications in 9.6% of cases, mostly in terms of nail dystrophies.^8^ The authors inferred a particularly high importance of conservative treatment in mild cases. However, proximal avulsion is preferred as the treatment of choice for more severe clinical presentations.^8^ It should be noted, however, that good patient education and regular follow‐up are essential for successful treatment after avulsion. Rehydrating nail bed care and daily massaging of the nail bed in the direction of nail growth prevent keratinization of the unprotected, delicate nail bed skin after avulsion and improve the postoperative results. Hypertrophy of the distal nail fold can be neutralized by taping (Figure 1e, f). In refractory cases or when patient cooperation is inadequate, a Howard‐Dubois procedure (“fish‐mouth plasty”) can be performed.^8^, ^10^ Our cases illustrate these two approaches and emphasize the importance of postoperative care for patients with retronychia.

## CONFLICT OF INTEREST STATEMENT

None.

## References

[1] de Berker DA , Richert B , Duhard E , et al. Retronychia: proximal ingrowing of the nail plate. J Am Acad Dermatol. 2008;58(6):978‐83. 10.1016/j.jaad.2008.01.013 18375016

[2] Baumgartner M , Haneke E . Retronychia: diagnosis and treatment. Dermatol Surg. 2010;36(10): 1610‐4. 10.1111/j.1524-4725.2010.01693.x 20722655

[3] Ventura F , Correia O , Duarte AF , et al. Retronychia‐clinical and pathophysiological aspects. J Eur Acad Dermatol Venereol. 2016;30(1):16‐9. 10.1111/jdv.13342 26435476

[4] Gerard E , Prevezas C , Doutre MS , et al. Risk factors, clinical variants and therapeutic outcome of retronychia: a retrospective study of 18 patients. Eur J Dermatol. 2016;26(4):377‐81. 10.1684/ejd.2016.2774 27122126

[5] Lencastre A , Iorizzo M , Caucanas M , et al. Topical steroids for the treatment of retronychia. J Eur Acad Dermatol Venereol. 2019;33(9):e320‐e322. 10.1111/jdv.15603 30932220

[6] Haneke E , Manola I , Smiljan Benko I , et al. Chronic Retronychia: Nonsurgical Treatment. Skin Appendage Disord. 2022;8(4):291‐294. 10.1159/000521496 PMC9274938 35983471

[7] Kim BR , Hur K , Lee SR , et al. Clinical features and treatment outcomes of retronychia: A multicenter retrospective analysis in Korea. J Am Acad Dermatol. 2022;87(1):221‐223. 10.1016/j.jaad.2021.07.053 34358584

[8] Sechi A , Zengarini C , Piraccini BM , et al. Treatment of retronychia: A systematic review and suggested treatment algorithm. Dermatol Ther. 2022;35(2):e15251. 10.1111/dth.15251 34877747

[9] Pessoa E Costa T , João AL , Lencastre A . Retronychia: A Paradigm Shift? Skin Appendage Disord. 2020;6(5):268‐271. 10.1159/000509370 PMC7548972 33088810

[10] Hassan RE , Khan L , Shah SH , et al. Surgical Strategies for Ingrown Toenails: A Comprehensive Review of Techniques, Outcomes, and Advancements. Cureus. 2024;16(1):e52501. 10.7759/cureus.52501 PMC10874229 38371148

